# The investigation of sudden arrhythmic death syndrome (SADS)—the current approach to family screening and the future role of genomics and stem cell technology

**DOI:** 10.3389/fphys.2013.00199

**Published:** 2013-09-12

**Authors:** Vishal Vyas, Pier D. Lambiase

**Affiliations:** ^1^Barnet and Chase Farm Hospitals NHS Trust, MedicineEnfield, UK; ^2^Cardiology, The Heart Hospital, University College Hospital and Institute of Cardiovascular Sciences, University College LondonLondon, UK

**Keywords:** sudden death, screening, ion channel, stem cell, SADS

## Abstract

SADS is defined as sudden death under the age of 40 years old in the absence of structural heart disease. Family screening studies are able to identify a cause in up to 50% of cases-most commonly long QT syndrome (LQTS), Brugada and early repolarization syndrome, and catecholaminergic polymorphic ventricular tachycardia (CPVT) using standard clinical screening investigations including pharmacological challenge testing. These diagnoses may be supported by genetic testing which can aid cascade screening and may help guide management. In the current era it is possible to undertake molecular autopsy provided suitable samples of DNA can be obtained from the proband. With the evolution of rapid sequencing techniques it is possible to sequence the whole exome for candidate genes. This major advance offers the opportunity to identify novel causes of lethal arrhythmia but also poses the challenge of managing the volume of data generated and evaluating variants of unknown significance (VUS). The emergence of induced pluripotent stem cell technology could enable evaluation of the electrophysiological relevance of specific ion channel mutations in the proband or their relatives and will potentially enable screening of idiopathic ventricular fibrillation survivors combining genetic and electrophysiological studies in derived myocytes. This also could facilitate the assessment of personalized preventative pharmacological therapies. This review will evaluate the current screening strategies in SADS families, the role of molecular autopsy and genetic testing and the potential applications of molecular and cellular diagnostic strategies on the horizon.

## Introduction

Of the 484,367 deaths registered in England and Wales in 2011, 29% were attributed to circulatory disorders (Births and Deaths in England and Wales, 2011). A report in 2009 found that sudden cardiac death was responsible for ~60,000 deaths, with ischemic heart disease the major cause (Papadakis et al., 2009). In the younger population (1–35 years of age), sudden cardiac death is the most common cause of premature death (Tester and Ackerman, 2012). Here, a thorough investigation including autopsy is critical in identifying a cause—for instance, structural abnormalities such as cardiomyopathies [e.g., hypertrophic cardiomyopathy (HCM)] may be identified at the point of autopsy. However, in a substantial proportion (reports ranging from 3–53% Tester and Ackerman, 2012) despite extensive post-mortem examination, no structural cause of death may be identified. It is thought that ion channelopathies such as Long QT Syndrome (LQTS) may explain a significant number of these events. Cases of sudden death in patients between the ages of 1–40 years with no previous cardiac history, who are seen alive in the 12 hours preceding death and have a normal coroner's autopsy (confirmation of a normal heart by an expert cardiac pathologist) in addition to a negative toxicological screen are deemed to have sudden arrhythmic death syndrome (SADS; Nunn and Lambiase, 2011). It is this syndrome that will form the focus of the review.

### Epidemiology

SADS is thought to be responsible for an estimated 0.24 deaths per 100,000 population according to an analysis of death certification data from England and Wales (Papadakis et al., 2009). This contrasts strongly with data available from northeast Thailand where it has been demonstrated that amongst men aged 20–49, the annual incidence of sudden unexplained death is 38 per 100,000 population per year (Tungsanga and Sriboonlue, 1993), with Brugada syndrome (BrS) thought to be the main cause for this dramatically higher rate of sudden death (Sangwatanaroj et al., 2001). Cardiac ion channelopathies such as LQTS, BrS, Catecholaminergic polymorphic ventricular tachycardia (CPVT) cannot be identified on conventional autopsy while certain cardiomyopathies including arrhythmogenic right ventricular cardiomyopathy (ARVC) and HCM may be missed at post-mortem due to subtle histological anomalies. The yield of genetic screening varies considerably according to the syndrome in question—some forms of LQTS e.g., Timothy Syndrome have a diagnostic yield that is near 100% while in ARVC, for instance, only 30–40% of clinically diagnosed patients have desmosomal mutations which are thought to be the main cause of the condition (Priori and Napolitano, 2006).

## Ion channelopathies associated with a structurally normal heart

The inherited arrhythmia syndromes LQTS, CPVT, and BrS form a substantial proportion of fatal arrhythmia-associated sudden deaths on the background of a structurally normal heart. Here, the epidemiology, genetic basis of the syndromes, the characteristic clinical features as well as insights into how genotype may play a role in guiding management and prognosis will be discussed (a list of known genes involved in the various syndromes is outlined in Table 1).

**Table 1:** **Genetic basis of principal ion channelopathies [adapted from Table 1, Giudicessi and Ackerman (2013)]**.

**Gene**	**Protein**	**Frequency**
**LQTS**		
*KCNQ1* (LQT1)	Kv7.1	30–35%
*KCNH2* (LQT2)	Kv11.1	25–30%
*SCN5A* (LQT3)	Nav1.5	5–10%
*ANKB* (LQT4)	Ankyrin B	<1%
*KCNE1* (LQT5)	MinK	<1%
*KCNE2* (LQT6)	MiRP1	<1%
*KCNJ2* (LQT7)	Kir2.1	<1%
*CACNA1C* (LQT8)	Cav1.2	<1%
CAV3 (LQT9)	Caveolin 3	<1%
*SCN4B* (LQT10)	Nav1.5 β4-subunit	<1%
*AKAP9* (LQT11)	Yotiao	<1%
*SNTA1* (LQT12)	Syntrophin- α 1	<1%
*KCNJ5* (LQT13)	Kir3.4	<1%
**CPVT**		
*RYR2* (CPVT1)	Ryanodine receptor 2	50-60%
*CASQ2* (CPVT2)	Calsequestrin 2	1–2%
*KCNJ2* (CPVT3)	Kir2.1	10%
**BrS**		
*SCN5A* (BrS1)	Nav1.5	20–30%
*GPD1L* (BrS2)	Glycerol-3-phosphate dehydrogenase 1-like	<1%
*CACNA1C* (BrS3)	Cav1.2	6.6%
*CACNB2* (BrS4)	Cav1.2 β2-subunit	<1%
*SCN1B* (BrS5)	Nav1.5 β1-subunit	<1%
*KCNE3* (BrS6)	MiRP2	<1%
*SCN3B* (BrS7)	Nav1.5 β3-subunit	<1%
*KCNJ8* (BrS8)	Kir6.1	2%
*CACNA2D1* (BrS9)	Cav1.2 α 2/∂1-subunit	<1%
*KCND3* (BrS10)	Kv4.3	<1%
*MOG1* (BrS11)	Mog1	<1%

*Note the likely syndrome subtype recorded in parenthesis but no consensus on numerical nomenclature for minor syndrome subtypes*.

### Long QT syndrome

**Figure 1:**
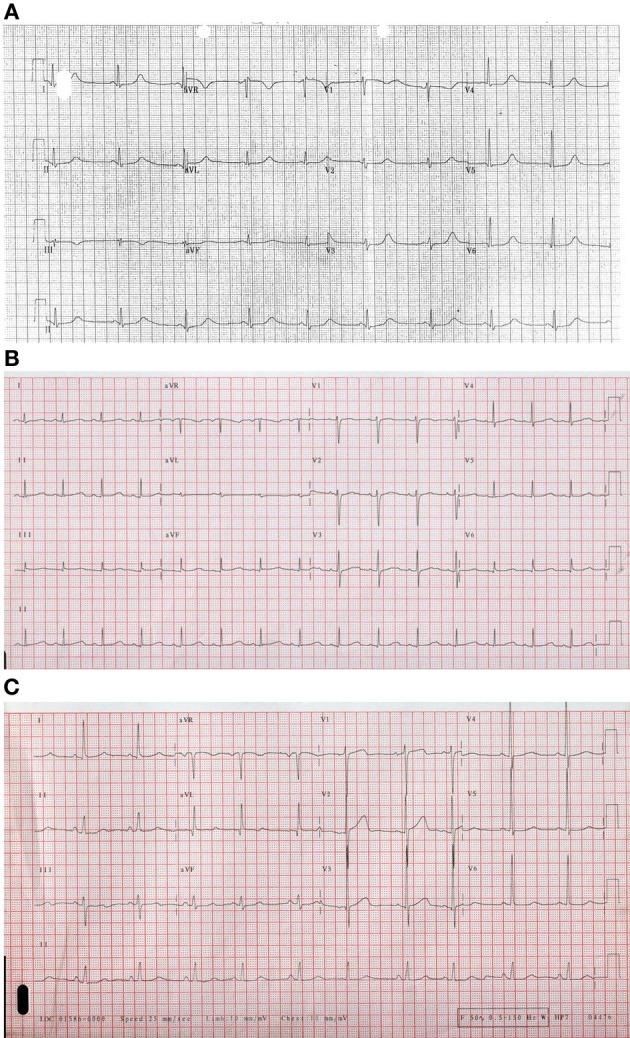
**Examples of ECGs for different forms of Long QT Syndrome (A)**. Long QT 1 **(B)** LQT2 illustrating bifid T wave morphology **(C)**. Long QT3 illustrating flat isoelectric segment with small symmetrical T wave particularly in lead III.

LQTS is defined by delayed repolarization of the myocardium. Clinically, this corresponds to a prolonged heart rate-corrected QT interval (QTc). Such patients have an increased risk of syncope, seizures, and sudden death. Its incidence is thought be 1/2000 (Schwartz et al., 2009) and so far 13 LQTS-associated genes have been implicated in this disorder. However, 60–75% of patients with definite LQTS have mutations in one of the three major susceptibility genes (Giudicessi and Ackerman, 2013). The remaining 10 genes are thought to increase the yield by less than 5% and contribute to an increase in false positives (Giudicessi and Ackerman, 2013). Triggers associated with LQTS include exertion, swimming, emotion, auditory stimuli e.g., alarm ringing with such triggers potentially bringing about syncope, seizures and in 5% untreated cases sudden fatal arrhythmia (Lambiase, 2010). Table 2 summarizes (Ackerman et al., 2011; Gollob et al., 2011; Tester and Ackerman, 2012; Giudicessi and Ackerman, 2013) the key features of the three principle LQTS syndromes and Figure 1 illustrates some typical ECG patterns.

**Table 2:** **Outline of the current genetic testing recommendations for the principal ion channelopathies**.

**Likely arrhythmia syndrome**	**Prevalence**	**Major clinical triggers**	**Characteristic ECG findings**	**Exercise testing**	**Pharmacological challenge testing**	**Recommended genetic testing**
LQT1	Overall LQTS 1/2000	Exercise	Broad tented T wave	QTc fails to shorten particularly in the recovery phase of exercise	IV epinephrine prolongs QTc (epinephrine could be helpful in unmasking LQTS and thus identifying new pathogenic mutations where the QTc is normal at rest)	*KCNQ1, KCNH2*, and *SCN5A*. Routine clinical testing of rare genes (<1% detection rate) not recommended. If patients have a clear clinical phenotype but negative in genetic testing for the above genes, rare genes may be assessed on a case-by-case basis.
LQT2		Emotional stimuli	Bifid T wave			
LQT3		Sleep and rest without arousal	Late onset peaked/biphasic T wave	Prolongation of QTc at night (24 hour ECG thus of great importance)		
BrS	1/2000	Rest or sleep	Coved-type ST-segment elevation in right precordial leads (type 1 ECG pattern)		IV sodium channel blocker converting a type 2 or type 3 ECG pattern to a type 1 ECG pattern	*SCN5A* gene testing only.
CPVT	1/7000–10,000	Emotion/Exercise (high adrenaline states)	Baseline usually normal	Bidirectional VT/polymorphic VT	IV infusion of adrenergic agonist inducing bidirectional VT/polymorphic VT	*RYR2* gene; if *RYR2* gene screening negative despite high clinical suspicion then consider *CASQ2* screening.

Current management guidelines of the Heart Rhythm Society/European Heart Rhythm Association (Ackerman et al., 2011) state that comprehensive/targeted LQTS genetic testing is recommended for anyone where there is strong evidence to suggest LQTS based on the phenotype (history, family history, ECG findings). For relatives of the index patient, mutation-specific testing is recommended even if they are asymptomatic with a normal ECG. Genotyping has been helpful in directing therapy: for instance β-blockade has been shown to have similar effects in preventing cardiac events in LQT1 and LQT2 patients but not having the same degree of beneficial effect in LQT3 (Moss et al., 2000).

### Brugada and early repolarization (“J wave”) syndrome

**Figure 2:**
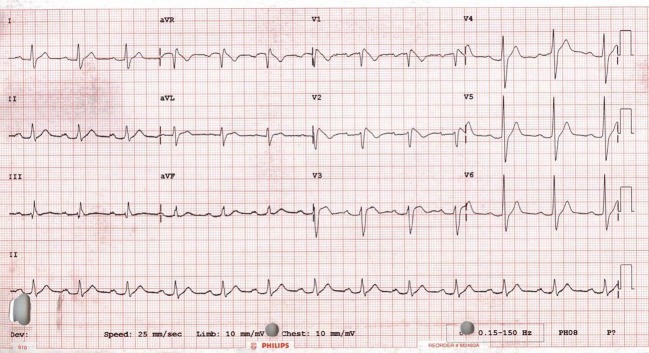
**A typical Brugada syndrome type 1 ECG showing coved ST elevation in leads V1 and V2**.

**Figure 3:**
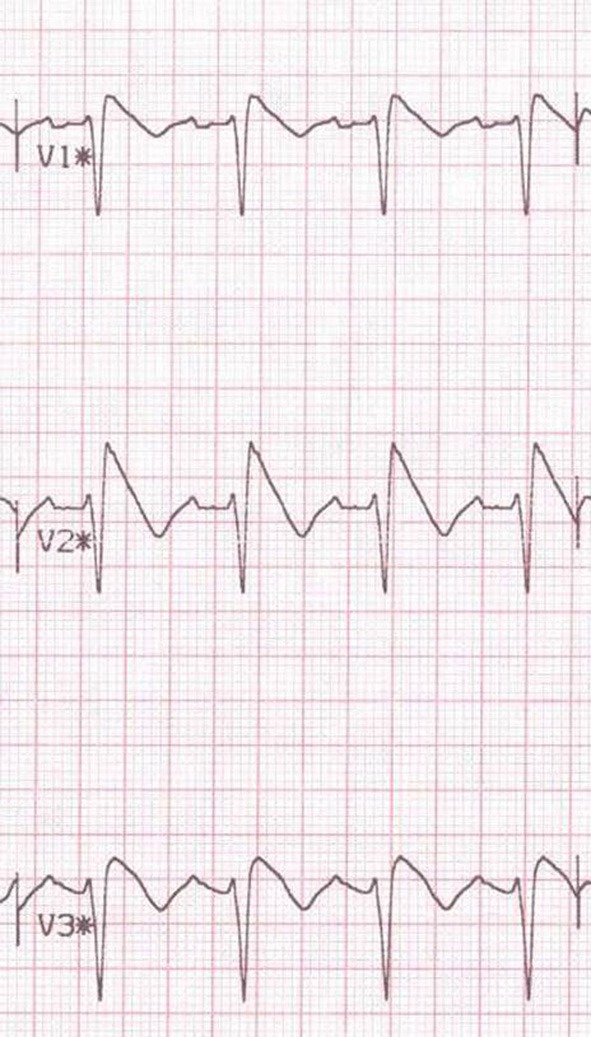
**Pharmacological challenge testing unmasking in Brugada phenotype**. Type 1 response to ajmaline challenge test.

BrS is characterized by coved-type ST-segment elevation followed by a negative T-wave in right precordial leads V1–V3 (type 1 ECG pattern) or saddleback pattern in V1 (with ST-segment elevation) or V2 (without ST-segment elevation). However, only a type 1 pattern is diagnostic of the syndrome where there is ST-segment elevation in >1 precordial lead (standard/high position V1–V3) in the presence/absence of a sodium channel blocker (see Figures 2, 3) in addition to either documented ventricular fibrillation (VF), polymorphic VT, family history of sudden cardiac death aged <45 years, coved-type ECG changes in family members, inducibility of VT with programmed electrical stimulation, syncope, or nocturnal agonal respiration (Haïssaguerre et al., 2008). The estimated prevalence is 1/2000 in Caucasians although the prevalence may be more in individuals of Asian descent (Antzelevitch et al., 2005). So far, mutations in at least 11 distinct susceptibility genes have been identified. The most common being mutations in the *SCN5A* gene (20–30% cases), while the other 10 genotypes identified so far are comparatively much rarer and thus only in BrS type 1 is gene testing for *SCN5A* mutations thought to be clinically useful at present, when there is a strong clinical suspicion of BrS based on clinical data (history, family history and ECG findings; Ackerman et al., 2011; Gollob et al., 2011). Cascade screening of relatives of the proband can follow thereafter. Patients are characteristically young males aged about 40 when the arrhythmias first manifest, with sleep being a major trigger (Antzelevitch et al., 2005). However, the majority of families screened have normal or borderline/subtle changes in the J point. Hence, pharmacological challenge testing using a sodium channel blocker is key in unmasking the condition (Figure 3).

Until recently inducibility of VT at electrophysiological study was thought to be predictive of sudden death risk (Priori et al., 2002a). However, recent large series have shown that this is not consistent, meaning it has dropped to a class IIb level of evidence in the recent HRS/EHRA Consensus Statement on the assessment of ion channel disorders (Eckardt et al., 2002; Tester and Ackerman, 2011; Priori et al., 2012). Other parameters such as ventricular effective refractory period, signal averaged ECG and ST changes in recovery may play a role in the future.

Currently, as per the HRS/EHRA guidelines, only *SCN5A* genetic testing is useful when there is strong clinical/ECG data to suggest BrS in the index case with a view to identifying a causative mutation and thereby facilitating cascade screening of relatives. However, as yet there is no clear therapeutic or prognostic utility in genetic testing (Ackerman et al., 2011).

Recent attention has been focused on the potential arrhythmogenic risk of J waves or early repolarization on the resting 12 lead ECG. Haïssaguerre et al. (2008) and Rosso et al. (2008) have demonstrated a significantly higher prevalence of J point elevation in the infero-lateral leads in idiopathic ventricular fibrillation survivors vs. matched healthy controls, coupled with pronounced J point elevation preceding the development of VF. The precise mechanism of this early repolarization phenomenon in humans remains uncertain. Recently, a Finnish population study of over 10,000 unselected people with a mean follow up of 30 years found that the presence of J point elevation was associated with a higher rate of death from cardiac causes and arrhythmia (Tikkanen et al., 2009) further supporting the notion that J point elevation may indicate an increased susceptibility to lethal arrhythmia, acting as a modifying factor whatever the underlying pathology. Our group has identified a higher proportion of SADS relatives with J waves vs. the general population, this suggests that the heritable J point elevation could account for a proportion of SADS deaths caused by ion channel mutations in the K_ATP_ channel and L-type Calcium channel (Antzelevitch and Yan, 2010; Nunn et al., 2011).

### Catecholaminergic polymorphic ventricular tachycardia (CPVT)

**Figure 4:**
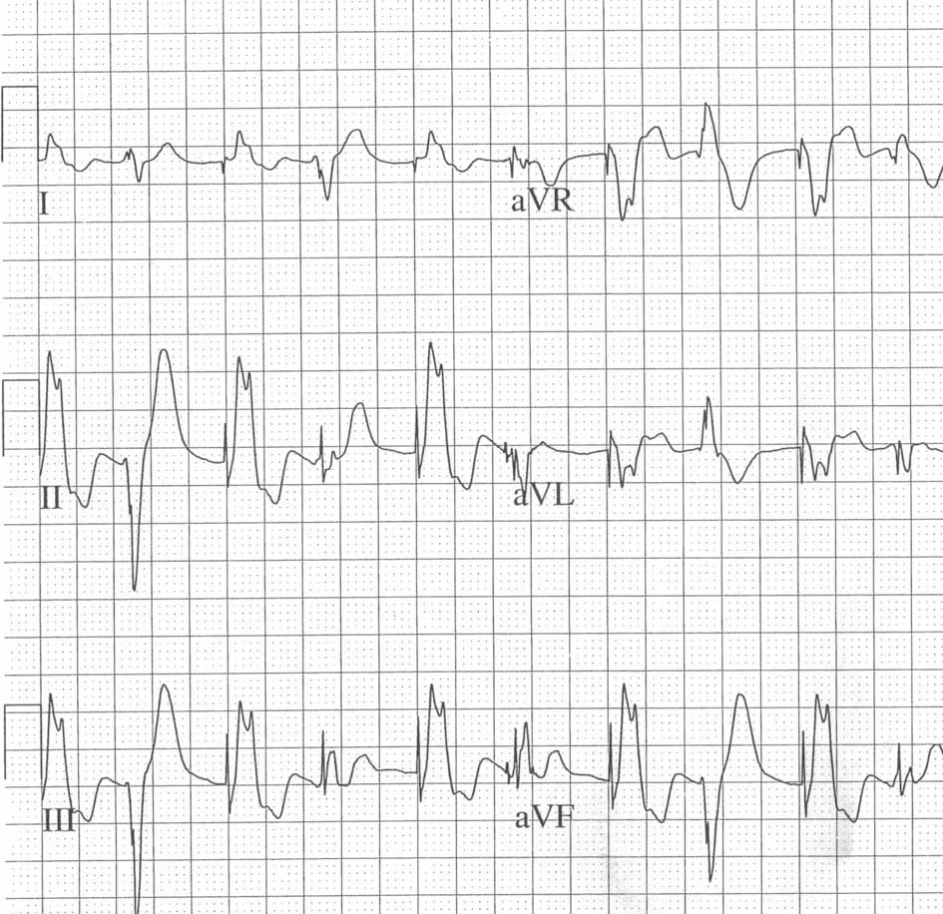
**Bidirectional VT in CPVT**.

CPVT is a disease of perturbed intracellular calcium homeostasis. Like LQTS, it is associated with a structurally normal heart however it usually displays a completely unremarkable resting ECG (occasionally bradycardia and U waves) and is usually only unmasked (ventricular ectopy is seen) following exertion or catecholaminergic stress testing (Tester and Ackerman, 2011) (Figure 4). It is thought to affect 1/7000–10,000 individuals with three distinct CPVT-associated genes so far identified (Giudicessi and Ackerman, 2013). Sixty to sixty-five percent of CPVT cases are associated with a mutation in the *RYR2*-encoded cardiac ryanodine receptor/intracellular calcium channel—CPVT type 1 (CPVT1; Priori et al., 2002b). The remainder of the mutations in the other two susceptibility genes are found in fewer than 5% cases (Crotti et al., 2012). A rare autosomal recessive form of CPVT has been associated with mutations in calsequestrin (*CASQ2*; Lahat et al., 2001). Both *CASQ2* and *RYR2* encode proteins involved in intracellular calcium handling. Mutations may predispose to elevated calcium levels during cardiac diastole and thus increasing the risk of developing ventricular arrhythmias. Genetic testing is currently recommended for anyone where clinical features and ECG findings observed on exercise/catecholaminergic testing indicate CPVT (Ackerman et al., 2011). This of course facilitates mutation-specific cascade screening of family members and advice on avoidance of triggers i.e., exercise. Genotype however does not impact on management or risk stratification strategies at present (Eckardt et al., 2002), although there is some evidence to indicate prophylactic β-blockade in these individuals may be useful.

### Short QT syndrome

This arrhythmia syndrome is comparatively infrequently encountered in clinical practice. It is however associated with a high incidence of syncope and sudden cardiac death, even in the younger patients and newborns. The defining electrocardiographic feature is QTc < 320 ms. Mutations in *KCNH2, KCNQ1*, and *KCNJ2* genes have been identified as associated with the condition. However, data is limited on the condition (a recent population screening study over 100,000 ECGs failed to identify a single case) and very little evidence is available to help guide genetic screening of this condition (Lambiase, 2010).

Having briefly overviewed the various arrhythmia syndromes, it is evident that a clinical diagnostic approach must first be followed in these scenarios and then targeted genetic screening if a clear phenotype is identified to maximize the yield of genetic screening in the family.

## Screening

### The clinical screening approach to SADS families

Screening of the 1st degree relatives in specialist clinics can diagnose an inherited cardiac condition in 22–53% of families. Behr et al. (2003) investigated 109 1st degree relatives of 32 people who died of SADS. Seven (22%) of the 32 families were diagnosed with an inherited cardiac disease. A subsequent more detailed evaluation of 57 consecutively referred families with SADS death identified 30 families (53%) with an inheritable heart disease (Giudicessi and Ackerman, 2013). Over half of those relatives affected received potentially lifesaving intervention with β-blockers and/or an ICD (Behr et al., 2008). Tan et al. (2005) investigated 43 consecutive families with >1 SADS victim who died at <40 years of age. Only 51% of probands underwent a post-mortem. 183 relatives were initially screened triggering the cascade screening of a further 150 relatives. A diagnosis was established in 17 of 43 families (40%) and revealed 151 pre-symptomatic disease carriers. Most recently, Wilde and colleagues (Van Der Werf et al., 2010) reported the assessment of 140 families with a sudden unexplained death (aged 1–50 years.) A diagnosis was established in 33% of families although a post mortem was performed in only 46% of cases. Strategies to increase the diagnostic yield include: ensuring all cases have a post-mortem, ideally with an expert cardiac pathologist review of the whole heart; reviewing as many 1st degree relatives in each family as possible; and carrying out a molecular autopsy where possible.

### Practical considerations of clinical screening

Effective clinical screening of SADS families requires a comprehensive multidisciplinary team approach. Moreover, chapter 8 of the National Service Framework in the UK requires the National Health Service (NHS) to provide a dedicated clinic to assess families with appropriately trained staff (Department of Health, 2005). A dedicated clinic typically comprises cardiologists, physiologists, echocardiographers, specialist nurses, bereavement and genetic counselors, and clinical geneticists (Lambiase, 2010).

Prior to seeing families in a SADS clinic, as much background information as possible should be obtained about the index case. This should include specific clinical information such as previous clinical encounters with a family physician or visits to the emergency department, as this may yield useful diagnostic information such as previous ECGs. Additionally, a detailed post-mortem report should be obtained. Usually a specialist nurse will coordinate the clinic and liaise with families, the family physician as well as the coroner's office to obtain relevant background information including a copy of the post-mortem report. Moreover, tissue sections or ideally the whole heart should be reviewed by a specialist cardiac pathologist to identify any histopathological evidence of an underlying cardiomyopathic process. For instance, myocyte disarray suggestive of HCM or local fibrosis and fatty infiltration indicative of ARVC may be identified (Lambiase, 2010).

Importantly, prior to conducting any investigations on SADS families, appropriate counseling must be undertaken to ensure the implications of the screening tests are well-understood. Employment, insurance, effects on children and other family members as well as the psychological impact of test results must all be appropriately discussed. The outcomes of investigations can vary from inconclusive non-diagnostic findings, which require regular follow-up to the need for medical therapy (e.g., β-blockers) or even invasive procedures such as the insertion of an implantable cardioverter-defibrillator (ICD) device (Lambiase, 2010).

## Consultation with physician

### History

Family screening should start with a detailed evaluation of the index case. The mode of death may provide useful information regarding etiology. For instance, long QT (LQT) 1 subtype (LQT1) and catecholaminergic polymorphic VT is associated with death while swimming (Tester and Ackerman, 2011). Syncope in the context of sudden loud noise or alarm is characteristic of LQT2 and death during sleep suggests the possibility of BrS and LQT3 (all three associated with an increased risk of arrhythmia during periods of increased cholinergic tone or bradycardia). Thus, a careful and systematic history from first-degree relatives can be great value in helping to determine the details prior to pre-syncopal or syncopal events as well as identifying other symptomatic family members.

Past medical history, previous accidents, prescribed/non-prescribed and recreational drugs e.g., cocaine use are all of relevance. Family history including any relatives who may have died under 40 years of age, family history of cardiac interventions e.g., pacemaker implantation, heart failure or any available death certification can all be invaluable. Certain markers in the history including syncope, 2 or more sudden deaths in the family at younger (≤40 years) age are all thought to be predictive markers of a diagnosis in the family (Tan et al., 2005; Behr et al., 2008).

### Investigations

A number of tests may be performed to aid in the screening process of SADS families. These will be at the physician's discretion and may vary between units. These can include a resting ECG, exercise ECG, VO_2_ max tests, signal averaged ECG, transthoracic echocardiogram and ajmaline or flecainide challenge test (to unmask BrS when suspected; Lambiase, 2010).

## The role of genetic testing

There has been a steady advance in the potential for gene testing since the discovery of the channelopathy-causing genes in the 1990s. With an ever-burgeoning panel of commercially available diagnostic genetic tests available to the electrophysiologist, consensus guidance is clearly highly relevant in helping to determine when and which tests are indicated. The Heart Rhythm Society/European Heart Rhythm Association Consensus Statement published in 2011 (Ackerman et al., 2011) aptly summarizes the current recommendations for genetic screening. These have been outlined together with overviews of the principal genetic syndromes associated with SADS in the summary table [Ackerman et al., 2011; Gollob et al., 2011; Tester and Ackerman, 2012; Giudicessi and Ackerman, 2013; above (Table 2)].

### Practical considerations of genetic testing

Genetic testing is usually employed as a confirmatory tool primarily to facilitate cascade screening of the family. Only after a thorough clinical assessment (with likely diagnosis established based on family history, patient background, ECG and pharmacological testing) should genetic screening be used in clinical practice. Moreover, genetic counseling (to include a comprehensive discussion of the relative risks and benefits) is highly recommended prior to any testing (Ackerman et al., 2011). The benefits offered by testing are in identifying a specific genetic mutation, which may help, to determine both management and prognosis of affected family members, best illustrated in the case of LQTS (see below). Alternatively, where the clinical screening process does not yield sufficient information to make a diagnosis or may require a large number of first-degree relatives to be screened, a single genetic test may prove to be the most cost-effective method. Furthermore, genetic testing may assist in family planning or for prenatal diagnosis in a parent who carries a known mutation (Schwartz et al., 2009). However, the yield of genetic testing varies considerably among the channelopathies; from 20% in BrS to 75% for LQTS (Ackerman et al., 2011). Hence, a negative test does not rule out the presence of a particular disease. Furthermore, the diagnostic, therapeutic and prognostic benefit derived from a genetic test is also highly disease-specific. Hence, testing must always be considered in the context of relevant clinical data (Ackerman et al., 2011).

### Clinical impact of genetic testing

Out of the channelopathies, LQTS testing has the highest yield as well as the maximum evidence for being able to guide diagnosis and management (Ackerman et al., 2011). Hence, this syndrome is well-placed to illustrate the clinical impact of genetic testing. Testing for the *KCNQ1, KCNH2*, and *SCN5A* genes, when LQTS is clinically suspected, should yield positive LQT1-3 causing mutations in 75% cases (Ackerman et al., 2011). However, as alluded to earlier negative testing does not exclude the disease where there is strong clinical suspicion. On the other hand, testing should not be performed without clinical suspicion, considering there is a substantial rate of rare variants of unknown significance (VUS) i.e., where there is insufficient evidence to label mutations as definitely disease-causing (discussed in detail later)—4–8% in the LQT1-3 genes (Kapa et al., 2009). Identifying a causative mutation in an index case of LQTS mandates mutation-specific cascade screening of all first-degree relatives (even when there is no clinical suspicion in that relative). Helpfully, in the absence of a clinical phenotype for LQTS in the relative, a negative genetic test will effectively exclude LQTS (Ackerman et al., 2011).

Genetic testing can additionally direct therapy in LQTS: β-blockers are highly protective in the case of LQT1 patients, moderately protective in LQT2 (Runa et al., 2008) while in LQT3 other agents such as mexiletine, flecainide, ranolazine, or propranolol are indicated (Schwartz et al., 1998; Moss et al., 2005, 2008; Ackerman et al., 2011). Hence, gene-directed therapeutic options are highly significant here. Prognostically, genotype appears also to be very significant: for instance, LQT1 transmembrane-localizing missense mutations have a greater risk of an LQT1-triggered cardiac event compared to a C-terminal mutation (Shimizu et al., 2004). In LQT2, patients with pore-region mutations tend to have longer QTc and more severe clinical manifestations (Moss et al., 2002). For the other principal channelopathies—CPVT and BrS, while cascade screening of index cases is indicated, there is no clear genotype-dependent differential therapeutic approach nor does genotype influence prognosis as yet (Ackerman et al., 2011).

In summary, genetic testing serves a vital role in confirmatory testing of individuals with a robust clinical phenotype and facilitates cascade family screening of index cases. However, at present, it is only in LQTS that genotype helps to clearly direct therapy and inform prognosis.

## Molecular autopsy: the new frontier

Molecular autopsy refers to the use of DNA extracted from tissue retained after the post-mortem, which can be utilized for confirmatory testing of a mutation identified in the relative (Lambiase, 2010). This approach facilitates post-mortem genetic testing of conditions that are known to cause SADS e.g., LQTS. The first reported case of the use of molecular autopsy to diagnose an inherited arrhythmia syndrome was by Ackerman et al. (1999). They found a 9 base pair deletion in the *KVLQT1* gene of a 19 year-old, previously fit and well lady, who had died after being resuscitated following a near-drowning experience. Subsequently, this same mutation had been found in the proband's other family members and appropriate therapy was then offered to the affected family members.

Only a handful of molecular autopsy series have been reported to date with one of the largest reported by Tester et al. (2012) who performed a comprehensive mutational analysis in 173 cases of the LQTS susceptibility genes as well a targeted analysis of the CPVT type 1-associated *RYR2* gene. Forty-five putative pathogenic mutations (25 novel mutations) were identified (26.0% yield). Correlation of genotype with phenotype demonstrated that females showing a higher yield than males (38.8 vs. 17.9%) and mutation-positive females were more likely to host an LQTS-associated mutation while mutation-positive males more likely to host a CPVT1-associated mutation. Exertion (34.8%) and sleep (18.6%) were also major triggers. Furthermore, 40.5% of cases (70/173) were found have a positive personal or family history of syncope, seizures, cardiac arrest, near drowning, or unexplained drowning (in a family member) or a known prolonged QT interval. This builds on their earlier study of 49 cases (Tester and Ackerman, 2007). Data from a series in Denmark (Larsen et al., 2013) found that in the 0–40 year old population, there was a yield of 8.3% in targeted *RYR2* gene sequencing. This follows-up from earlier series including that by Chugh et al. (2004), who showed in a cohort of 270 sudden death cases over a 13 year period that there were 12 autopsy negative cases of which 2 showed a mutation in the *KCNH2* gene. More recently, Skinner et al. (2011) found that in a prospective population-based long QT molecular autopsy study of 1–40 year-olds, 5/33 had rare possible LQTS-associated mutations.

### Practical considerations

In most autopsy studies, DNA extraction is typically based on formalin-fixed, paraffin-embedded tissue (FF-PET) due to the comparative ease of storing and transporting tissue (Basso et al., 2010). However, DNA extracted from this source is thought to be unreliable for molecular autopsy and usually inadequate for comprehensive post-mortem genetic testing (to detect a pathological mutation; Carturan et al., 2008). Indeed, a study by Doolan et al. (2008) found no putative pathogenic mutations in a series of 59 cases of sudden expected death when using DNA extracted from FF-PET. Optimal sources of intact DNA include blood collected in ethylenediaminetetraacetic acid (EDTA) or frozen heart, liver, or spleen tissue. Furthermore, 10–15 ml of EDTA blood or 5–10 g of fresh tissue should be obtained at autopsy and stored at −80°C to provide the ideal source of material for comprehensive genetic testing (Ackerman et al., 2001). In order to try and combat the limitations of material for autopsy, Gladding et al. (2010) used DNA extracted from Guthrie blood spots and used whole genome amplification prior to sequencing. They found out of 19 cases in their series, 4 had pathological mutations and all probands had at least one first-degree family member with the same mutation. This was followed-up by a Danish group (Winkel et al., 2012) who showed a yield of 11% for 3 major LQTS-associated genes amongst a cohort of 1–35 year-olds.

The advent of full exonic sequencing will mean that the whole patient exome can be sequenced. This significantly reduces the cost of gene testing but also generates enormous quantities of bioinformatic data with multiple genetic variants and potentially mutations in other non-cardiac genes being identified. At present, we only have a limited understanding of the etiology of recognized arrhythmic conditions. Hence, the interpretation of any additional genetic information must be carefully assessed and put into the context of the patient/family being screened. This is particularly important considering we are currently severely hampered by our limited ability to assess the pathogenicity of specific variants.

### Recommendations

Most of the series published so far are a relatively small in size. Thus, larger studies and analyses are required to help better characterize the yield of mutation detection and also offer better phenotype/genotype correlations. For instance, helping to identify clinical correlations between genetic mutations and clinical characteristics such as age or gender. This would contribute to guiding the clinical evaluation of the proband's family members and improving the cost effectiveness of the current approach. At present a combined clinical diagnostic approach and molecular autopsy would be recommended, as mutation carriers may only have minor manifestations of disease and compound heterozygotes may have clinically milder or more severe forms of the condition depending upon the functionality of the complementary allele gene. For example, if the complementary gene is itself a polymorphism with a down-regulation in function to 30%, this cannot compensate for the effects of a non-functioning channel and hence significantly reduces repolarization reserve in the case of LQTS (Crotti et al., 2005).

## Variants of unknown significance

Another major question looms regarding VUS identified on gene testing. As briefly mentioned earlier, VUS refer to mutations where there is inadequate evidence to deem them as disease-causing; increasingly, this is becoming an issue with the declining costs of genetic screening allowing large sections of the genome or indeed the whole exome to be sequenced. Cotton and Scriver (1998) have outlined several criteria that help determine whether a mutation or variant is indeed a disease-causing mutation (when taken in context of the other clinical data). These include:

– non-sense/frameshift mutations leading to generation of stop codons or downstream stop codons, respectively
– insertion/deletion mutations leading to truncated protein products
– co-segregation of the variant with disease
– absence/rarity of the variant in control populations
– mutations in highly conserved amino acid residues/domains likely altering the gene product
– functional analysis of the gene product through *in vitro* expression analysis

Satisfying any one of the criteria above does not necessarily point toward a definitive designation as a pathogenic mutation. Moreover, if taken together the criteria above do not allow for a variant to be considered disease-causing, it is thereafter deemed a VUS until further analysis (including functional assessment) is used to confirm whether it is pathogenic. Moreover, the presence of a VUS should not be used to assist in the diagnosis of an index patient nor should it be used for cascade screening of relatives (Giudicessi and Ackerman, 2013).

In light of recent genome-wide association studies (GWAS), new insights have been gained into potential variants that may be associated with increased risk of sudden cardiac death. Arking et al. (2006) assessed the QT interval extremes in a cohort of German subjects. They identified *NOS1AP*, a regulator of neuronal nitric oxide synthase as a potential novel disease-causing gene modulating cardiac repolarization with one minor allele explaining up to 1.5% of QT interval variation. Further studies specifically assessed the effect of *NOS1AP* variants in known ion channelopathy populations. Crotti et al. (2009) assessed the clinical manifestations and symptom occurrence in a South African LQTS population (with a *KCNQ1* mutation). They found that LQTS individuals with the rs4657139 variant in *NOS1AP* had greater probability of cardiac arrest and sudden death and had a greater likelihood of having a more prolonged QT interval. Taken together these findings would indicate that variants in the *NOS1AP* gene could act as genetic modifiers with a potentially significant impact on electrical function. Tomas et al. (2010) findings supported the work of Crotti et al. (2009). They found in a LQTS1 cohort with mutations in 5-associated genes that alleles rs4657139 and rs16847548 were associated with an increased risk of cardiac events. This would add further evidence to the notion that specific variants in *NOS1AP* gene act as risk-modifiers in known LQTS patients.

Albert et al. (2010) demonstrated in a case-control study of 6 prospective cohorts that 2 common intronic variants in the *KCNQ1* and *SCN5A* genes were significantly associated with sudden death in individuals of European descent (after adjustment for cardiovascular risk factors). The alleles identified were the T-allele at rs22832222 in *KCNQ1* with a population frequency of 67 and 60% for the C-allele at rs11720523 in the *SCN5A* gene.

Finally, in the largest meta-analysis of GWAS to date, Arking et al. (2011) found that there is a strong association with sudden cardiac death at locus 2q24.2 including the *BAZ2B* gene, which is thought to increase risk of sudden cardiac death by >1.9 fold per allele in individuals of European descent.

While GWAS have opened an avenue to identifying novel VUS which may indeed go on to be causative or risk-modifying due to the impact on electrical function, caution must be exercised until further functional studies are performed to identify the risk of cardiac events. Hence, the presence of a VUS should not be used to assist in the diagnosis of an index patient nor should it be used for cascade screening of relatives at present and is therefore, as yet, generally not a realistic screening tool (Giudicessi and Ackerman, 2013).

## The potential role of induced pluripotent stem cell technology in functional ion channel mutation testing and clinical evaluation

Since human embryonic stem cells (hESCs) were first isolated from blastocysts in 1998, it has become possible to produce human-induced pluripotent stem cells (hiPSCs) by reprogramming somatic cells with just four genetic factors (Thomson et al., 1998; Takahashi et al., 2007; Yu et al., 2007). This means that a skin biopsy can be taken from a patient or SADS index case and fibroblasts cultured and reprogrammed to create specific cell lines with cardiomyocytes being the most relevant in the context of SADS.

**Figure 5:**
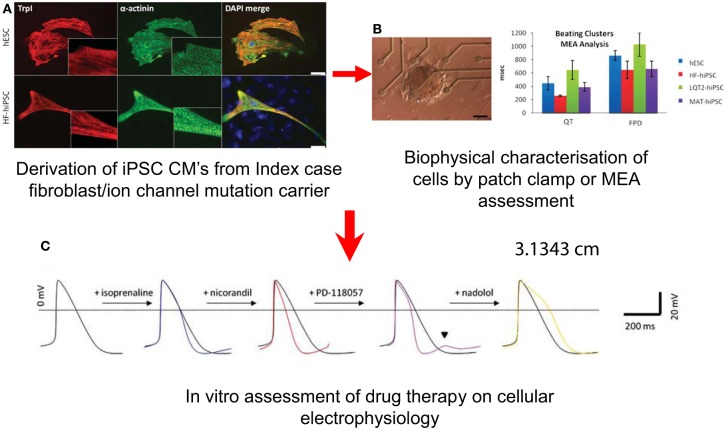
**Potential Uses of iPSC-CMs in the Investigation of SADS**. Derivation of cells **(A)** Immunostaining for cardiac troponin I (TrpI) and a-actinin in myocytes derived from hESCs, HF–hiPSC, LQT2–hiPSC, and MAT–hiPSC, showing characteristic cardiac muscle striations. **(B)** Characterization of cellular electrophysiology: Image of an LQT2–hiPSC beating cluster mounted onto a multi-electrode array for electrophysiology analysis, and graph showing prolongation in QT interval and field potential duration in LQT2–hiPSC beaters relative to controls. (From Matsa et al., 2011). **(C)** Testing of drugs: (i) Averaged and (ii) raw action potential curves of LQT2–hiPSC myocytes showing isoprenaline-induced arrhythmogenesis (blue traces) and attenuation of this phenotype by nadolol (yellow trace) or propranolol (green trace).

These cells can be characterized by techniques including patch clamping and multi-electrode array (MEA) to interrogate their electrophysiological behavior (Terrenoire et al., 2013) (Figure 5). Alterations in calcium handling can be visualized using real-time microscopy utilizing calcium sensitive dyes (Jung et al., 2011). There is now data from hiPSC lines carrying mutations that cause LQTS and CPVT which shows these cells not only recapitulate the clinical phenotypes but the response to drugs can be reproduced *in vitro* (Figure 5) (Matsa et al., 2011). This has an advantage over heterologous cell expression systems for testing individual ion channel mutations using human embryonic kidney (HEK) cells, and Chinese Hamster Ovary (CHO) cells which lack the ion channels and cofactors that are relevant to human cardiac electrophysiology. In the case of LQTS2, caused by mutations in the I_Kr_ channel, hiPSC-derived cardiomyocytes (hiPSC-CMs) developed arrhythmias when exposed to isoprenaline, a stressor used clinically to precipitate and diagnose the condition (Terrenoire et al., 2013). This effect could be reversed by applying the patient's own medication, nadolol (a β-blocker), dantrolene and roscovitin; drugs known to be beneficial in moderating calcium flux, stabilized ion flux in hiPSC models of the calcium channel disorders, CPVT and Timothy syndrome (linked to LQT type 8), respectively (Matsa et al., 2011; Pasca et al., 2011; Yazawa et al., 2011).

However, this approach still requires significant refinement as there are a number of issues related to iPSC technology which will affect its application to the evaluation of specific ion channel mutations. The utilization of this technology and its pitfalls has been recently extensively reviewed in this journal (Hoekstra et al., 2012). Mixed cultures of atrial and ventricular myocytes are obtained affecting cellular electrophysiology and their response to specific drugs. These selected populations of the derived cells vary in their membrane potentials compared to other populations, which will affect the ion channel gating properties and thereby alter the behavior of a specific mutant ion channel under investigation. The cells often manifest a more immature electrophysiological phenotype. While I_to1_, I_K1_, and I_f_ are present in hiPSC-CMs (Ma et al., 2011), their contribution to the biophysics of the cell has not been verified. The presence of I_NCX_ has not been studied in detail but its functional presence can also be presumed since intact calcium handling has been demonstrated (Itzhaki et al., 2011; Lee et al., 2011). Currently, there is no evidence for the functional presence or absence of I_K,ACh_. The functional presence of sarcoplasmic reticulum (SR), ryanodine receptors (RyRs), and the calcium-binding protein CASQ2 has been demonstrated (Itzhaki et al., 2011; Lee et al., 2011; Novak et al., 2012). However, due to the absence of t-tubulin in hiPSC-CMs, the coupling between calcium influx through L-type calcium channels and calcium release from the SR through RyRs is significantly reduced. Therefore, the use of hiPSC-CMs to study specific cardiac arrhythmia syndromes influenced by calcium handling e.g., CPVT and LQT8, has to be focused on the biophysical properties of the affected protein. Some of these issues can be addressed by generating and assessing more refined populations of iPSCs in order to ensure that they have more consistent electrophysiological profiles to faciliate mutant protein profiling and testing of drugs.

Taking into account the technical limitations and current caveats of utilizing iPSC technology, this approach has potential value in the field of SADS on a number of fronts. First of all in the case of an ion channel VUS, the behavior of that channel could be assessed in the patients' derived cardiomyocytes to assess the extent to which it causes significant alterations in ion channel function, trafficking and action potential duration. Furthermore, in SADS victims or idiopathic VF survivors where no known disease causing mutation has been identified, the function of specific ion channels and cellular electrophysiology could be interrogated to identify novel causes of lethal arrhythmia and new drug targets. The specific problem of compound heterozygosity where more than one VUS is inherited, which may only partially down-regulate ion channel function/trafficking can also be evaluated in the individual patient. Therefore, one can assess if this particular combination of defects actually exerts important cellular electrophysiological abnormalities, which are pro-arrhythmic and not necessarily manifest on routine clinical testing. This information could be utilized to prescribe prophylactic drug therapy and protect against cardiac arrest in at-risk individuals. This concept has recently been illustrated when hiPSCs were produced from a healthy donor as well as from a mother and daughter, wherein the mother was clinically asymptomatic with a moderately prolonged QT interval and the daughter was symptomatic with an excessively prolonged QT interval (arrhythmias, syncope, and seizure episodes). Recording action potential durations from the different hiPSC-cardiomyocytes showed that the clinical profile was reflected *in vitro* (i.e., action potential longest in the daughter's cells, then the mother's, then the healthy control) and only hiPSC-cardiomyocytes produced from the daughter developed spontaneous arrhythmias (Terrenoire et al., 2013). The next decade should help establish whether *in vitro* to *in vivo* associations can be applied in other conditions with important mechanistic and therapeutic implications in this and other arenas.

## Conclusions

**Figure 6:**
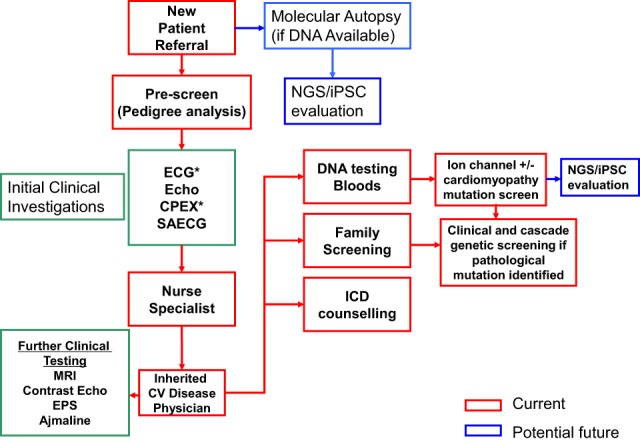
**Overview of the different strategies in the management of a SADS family**. Abbreviations include: EPS, electrophysiological study; Echo, echocardiogram; CPEX, cardiopulmonary exercise testing; SAECG, signal averaged ECG; NGS, next-generation sequencing.

The diagnosis and management of SADS families is evolving rapidly (Figure 6 summarizes the current management approaches). Comprehensive clinical assessment is still the mainstay of screening families of SADS patients with highly specific recommendations for complementing clinical assessment with genetic screening options. Molecular autopsy adds another strategy in the armory of screening of SADS families and validating cases of SADS. However, with a limited understanding of the etiology of the heritable arrhythmic syndromes, careful interpretation of the added genetic information in the context of the family being screened is critical. VUS are as yet not a viable screening tool but provide a burgeoning data set to validate through functional studies. Finally, the new techniques of full exonic sequencing and iPSC technologies will certainly facilitate diagnosis and management. However, the clinical relevance of mutation screening and iPSC-derived information will need to be carefully translated back to the individual relatives, in order to ensure that any anomalies identified are clinically relevant and impact on the patient's well-being and arrhythmogenic risk.

### Conflict of interest statement

The authors declare that the research was conducted in the absence of any commercial or financial relationships that could be construed as a potential conflict of interest.
